# A retrospective comparison of inappropriate prescribing of psychotropics in three Norwegian nursing homes in 2000 and 2016 with prescribing quality indicators

**DOI:** 10.1186/s12911-019-0821-0

**Published:** 2019-05-29

**Authors:** Jan Schjøtt, Jörg Aßmus

**Affiliations:** 1 Department of Clinical Biochemistry and Pharmacology, Haukeland University Hospital, 5021 Bergen, Norway; 2 Department of Clinical Science, Faculty of Medicine and Dentistry, University of Bergen, Bergen, Norway; 3 Centre for Clinical Research, Haukeland University Hospital, Bergen, Norway

**Keywords:** Drug interactions, Inappropriate prescribing, Medical informatics, Nursing homes, Psychotropics

## Abstract

**Background:**

Inappropriate prescribing of psychotropics is a persistent and prevalent problem in nursing homes. The present study compared inappropriate prescribing of psychotropics in nursing homes 16 years apart with prescribing quality indicators. The purpose was to identify any change in inappropriate prescribing of relevance for medical informatics.

**Methods:**

Three Norwegian nursing homes were audited in 2000 and 2016 with regard to prescribing quality. Psychotropics among 386 patients in 2000, and 416 patients in 2016, included combinations of antidepressants, antipsychotics, anxiolytics-hypnotics, and antiepileptics. Prescribing quality indicators included psychotropic polypharmacy (defined as concurrent use of three or more psychotropics) and potential inappropriate psychotropic substances or combinations. Furthermore, potential clinically relevant psychotropic interactions were classified as pharmacodynamic or pharmacokinetic using an interaction database. The first ranked (most important) interaction in each patient was selected with the following importance of categories in the database; recommended action > documentation > severity. Three levels (from low to high) within each category were used for ranking.

**Results:**

From 2000 to 2016, psychotropic polypharmacy increased from 6.2 to 29.6%, potential inappropriate psychotropic substances was reduced from 17.9 to 11.3% and potential inappropriate psychotropic combinations increased from 7.8 to 27.9%. Changes in polypharmacy and combinations were predominantly associated with prescribing of anxiolytics-hypnotics. Sixty-three patients (16.3%) had psychotropic interactions in 2000 increasing to 146 patients (35.1%) in 2016. The increase in interactions was associated with prescribing of antidepressants. First ranked interactions, more than 60% of all interactions in both years, were increasingly pharmacodynamic, from 69.9 to 91.0%. Interactions in 2016 were associated with a lower level of recommended action and documentation, but not severity compared to 2000. The inappropriate prescribing of antipsychotics and antiepileptics was reduced in 2016 compared to 2000.

**Conclusions:**

Using prescribing quality indicators we observed the importance of antidepressants and anxiolytics-hypnotics for inappropriate prescribing in 2016 while the role of antipsychotics and antiepileptics were reduced compared to 2000. A change to mainly pharmacodynamic interactions that lack good documentation was also observed. The present findings can be used for medical informatics-based approaches to address specific problems with prescribing, and prescribing quality indicators, in Norwegian nursing homes.

## Background

Use of one or more psychotropic drugs (e.g. psychotropics like antidepressants, antipsychotics, anxiolytics and hypnotics among others) is common in nursing homes [1–3]. Use of psychotropics is high among patients with dementia (about 80% of nursing home patients), and psychotropics are used in treatment of agitation, delusions, anxiety, depression, and sleep problems [4, 5]. Inappropriate treatment with multiple psychotropics has been identified as the most frequent drug-related problem in nursing homes [6, 7]. A recent cross-sectional analysis among 451 nursing homes in France found that polypharmacy and prescribing of potentially inappropriate medications mainly concerned psychotropics [8]. Psychotropics are associated with several adverse effects in older adults, most importantly risk of fall injuries, hospitalisations and mortality [9, 10].

Prescribing quality indicators are recommended to support clinical decision making in pharmacotherapy [11]. Indicators for inappropriate use of psychotropics include “concurrent use of three or more psychotropics” [12]. This indicator is considered to be a proxy for polypharmacy with risk of adverse effects and psychotropic interactions (PIs). Potentially inappropriate psychotropic combinations (PICP) or potentially inappropriate psychotropic substances (PIPS) are more specific indicators compared to “concurrent use of three or more psychotropics”, particularly with respect to increased risk of adverse events among the elderly [12]. Drug interaction databases are also frequently used to assess prescribing quality in nursing homes [7, 8]. Considering the quality of medical informatics and decision support, subscription databases should be preferred to open access databases or prescribing and dispensing software systems [11, 13, 14].

The present study compared prescribing of psychotropics in 2000 and 2016 with prescribing quality indicators. The purpose was to identify any change in inappropriate prescribing of psychotropics with regard to polypharmacy, PICP, PIPS, and PIs. Inappropriate prescribing of psychotropics is a persistent and prevalent problem in nursing homes in Europe despite the introduction of several prescribing quality indicators [6–8, 15]. Identification of current and historical problems with inappropriate prescribing, and prescribing quality indicators, could be useful for medical informatics-based approaches in Norwegian nursing homes.

## Methods

### Study population

One-day, point-prevalence of psychotropic prescribing in a convenience (non-randomised) sample was collected from the same three nursing homes in Bergen, Norway 16 years apart. No clinical or personal information other than patients’age and gender was collected. Nor was information pertaining to physicians, other staff, or details concerning organisation of the nursing homes. Only patients’aged ≥ 65 years were included at both sampling times. Researchers were blinded to patients’nursing home residence in 2016.

The three nursing homes in 2016 included regular units that provided long-term care, respite care, and rehabilitation as well as units for short-term care. They also included special care units that provide sheltered care for patients with dementia and psychogeriatric diseases. Thus, they reflect the transition in nursing homes from 2000 to 2016 with institutions consisting of mainly regular units to institutions that also provide various more specialized units. The two audits of prescribing were regarded as representative for identifying inappropriate prescribing of psychotropics.

### Psychotropics

The information on prescribing of psychotropics was manually collected from medical charts (cardex) by pharmacists in March 2000. Data included prescribed psychotropics, time of administration and respective doses. In March 2016, similar data were retrieved automatically from electronic charts. Psychotropics were classified according to the Anatomical Therapeutic Chemical (ATC) classification system [16], and included antipsychotics (ATC-code N05A), anxiolytics (N05B), hypnotics (N05C), antidepressants (N06A) and antiepileptics (N03A). Anxiolytics and hypnotics were collapsed into one category (anxiolytics-hypnotics) based on the assumption that the two categories have comparable adverse effects among nursing home patients. Antiepileptics are increasingly prescribed for psychiatric indications in Norway, and epilepsy increases with age [17]. Of relevance for inappropriate prescribing of psychotropics, data concerning all prescribed psychotropics (regularly prescribed and additional drugs as needed) were included [18]. Concurrent use of psychotropics from a similar ATC category is not uncommon in nursing homes, but resulted in a single registration for each patient (e.g. the patient uses ≥ 1 antidepressants).

### Polypharmacy, PICP and PIPS

Polypharmacy was defined as concurrent use of three or more psychotropics [12]. PICP were defined as combinations of two or more psychotropics from the same ATC-category. PIPS were defined as long-acting benzodiazepines and psychotropics with anticholinergic effects [12]. PICP were defined from the third level (pharmacological subgroup; e.g N05A, antipsychotics), and polypharmacy and PIPS were defined at the fifth level (chemical substance; e.g. N05A, risperidone) in the ATC-classification system [16]. The prescribing quality indicators above are recommended for patients aged 75 years or older, but could be relevant for younger patients (65 years or older) in nursing homes, and were used accordingly in the present study.

### PIs

We also assessed the most important PIs in the prescription data of the respective years. The first ranked PI in a patient was selected by use of the subscription interaction database Stockley’s Interaction Alerts (SIA) [19]. SIA provides consistent albeit briefer information on drug interactions compared to Stockley’s Drug Interactions, and describes classification of the clinical relevance of a drug interaction. A clinically relevant PI in SIA is classified with the following three categories: recommended action, severity, and documentation. In SIA, the recommended action for a clinically relevant PI is either “informative”, “monitor”, “adjust dose”, or “avoid”. *Recommended action* for a clinically relevant PI in the present study was defined by collapsing “monitor” and “adjust dose” into the following three levels (from low to high); “informative”, “monitor or adjust dose”, or “avoid”. In the present study, *severity* was classified (from low to high) as “mild”, “moderate” or “high” in concordance with SIA. Documentation in SIA is classified with four levels; “theoretical”, “case”, “study” or “extensive”. “Extensive” is an option for documentation of interactions where the information provided is based on numerous small or medium size studies or several large studies usually supported by case reports [19]. *Documentation* of a clinically relevant PI in the present study was defined by collapsing “study” and “extensive” to aquire the following levels (from low to high); “theoretical”, “case”, or “study”. The first ranked PI in each patient was defined by the following hierarchy of categories and levels; recommended action (avoid > monitor or adjust dose > informative) > documentation (study > case > theoretical) > severity (severe > moderate > mild). The order of the categories were based on our experience that many PIs may be described as potentially severe, but recommended action (e.g. contraindicated) and documentation (study) is of importance when providing drug information. The number of concurrent PIs was registered for each patient. A clinical pharmacologist (JS) defined each PI as pharmacodynamic or pharmacokinetic based on the description in SIA. If a pharmacokinetic PI contained an additional description of a pharmacodynamic effect which could not be explained by a change in plasma level of one or both drugs, the PI was defined as pharmacokinetic.

### Outcomes and predictors

The study investigated the appearance of different types of psychotropics as well as their combinations and interactions as outcomes. Predictors of having ≥ 1 PIs among patients with ≥ 2 concurrent psychotropics included age, gender, number of psychotropics, and use of antidepressants, antipsychotics, anxiolytics-hypnotics or antiepileptics. Dichotomous variables were coded as 0 or 1 for absence or presence of a quality (e.g. no use of antidepressants = 0; use of ≥ 1 antidepressants = 1). Age and number of psychotropics were introduced in the model as continuous variables.

### Statistical analysis

Comparison of continuous variables including age and number of psychotropics or PIs was performed by the Mann-Whitney *U*-test. Comparisons of proportions was performed by Chi-square test or Fischer’s Exact test. The association between the predictors and the appearance of PIs was assessed using a logistic regression model in three steps. In the first step we estimated the univariate model for each predictor and in the second we estimated the fully adjusted model containing all predictors. In the third step we defined the final model including only predictors with a p-value < 0.25 in one of the first steps. Predictors were examined for multicollinearity by linear regression analysis. P-values < 0.01 were accepted as statistically significant. SPSS version 24 (IBM Corp, Armonk, NY) was used for data analysis.

## Results

### Study population

In 2000 there were 386 patients in the three nursing homes increasing to 416 in 2016. The proportion of male residents increased from 26.4% to 33.9% in 2016. Mean age ± standard deviation (SD) among patients was 84.6 ± 7.2 years in 2000, and 84.3 ± 8.1 years in 2016 (p = 0.704). Women were older than men at both sampling times, but there were no significant age differences between the time-points within each gender. There were no significant differences with regard to age or gender among subgroups of patients who used psychotropics or had PIs. No patients were younger than 65 years in 2000. Twenty patients aged 23 to 64 years were excluded from the material in 2016.

### Psychotropics

Twenty-two different psychotropics were prescribed in both 2000 and 2016, while 14 and 13 psychotropics were unique for 2000 and 2016, respectively (Table 1). Relatively few patients (< 5%) were prescribed unique drugs in 2000. Three of the unique drugs in 2016 were prescribed to > 10% of the patients. The five most frequent psychotropics prescribed to patients in 2000 were citalopram, oxazepam, zopiclone, risperidone and carbamazepine. In 2016, the most frequent prescribed were oxazepam, zopiclone, mirtazapine, quetiapine and escitalopram. Among psychotropics prescribed in both 2000 and 2016, the increase of oxazepam and zopiclone was most striking. In 2000, 386 patients were prescribed 352 psychotropics compared to 416 patients and 765 psychotropics in 2016. On average, 0.91 psychotropics per person in 2000 increased to 1.84 in 2016 in the three nursing homes. The median number of concurrent psychotropics was two in both years. A range of two to four concurrent psychotropics in 2000 increased to two to six in 2016.

**Table 1:** Psychotropics prescribed only in 2000, in both years, and only in 2016 in three nursing homes with 386 and 416 patients the respective years

	2000		2000 and 2016			2016	
	Drug	Patients n (%)	Drug	Patients n (%)	Patients n (%)	Drug	Patients n (%)
Patients		386 (100.0)		386 (100.0)	416 (100.0)		416 (100.0)
Antidepressants	Clomipramine	1 (0.3)	Amitriptyline	8 (2.1)	2 (0.5)	Bupropione	4 (1.0)
	Doxepine	2 (0.5)	Citalopram	51 (13.2)	25 (6.0)	Escitalopram	57 (13.7)
	Nortriptyline	6 (1.6)	Mianserin	15 (3.9)	10 (2.4)	Mirtazapine	89 (21.4)
	Paroxetine	16 (4.1)	Sertralin	8 (2.1)	15 (3.6)	Moclobemide	1 (0.2)
						Venlafaxine	11 (2.6)
Antipsychotics	Chlorpromazine	7 (1.8)	Chlorprotixene	2 (0.5)	3 (0.7)	Clozapine	4 (1.0)
	Flupenthixol	3 (0.8)	Haloperidol	14 (3.6)	27 (6.5)	Quetiapine	72 (17.3)
	Fluphenazine	1 (0.3)	Levomepromazine	18 (4.7)	4 (1.0)		
	Melperon	5 (1.3)	Lithium	2 (0.5)	1 (0.2)		
	Perphenazine	3 (0.8)	Olanzapin	7 (1.8)	13 (3.1)		
	Sertindole	1 (0.3)	Risperidon	33 (8.5)	8 (1.9)		
			Prochlorperazine	2 (0.5)	1 (0.2)		
			Zuclopentixol	4 (1.0)	2 (0.5)		
Anxiolytics-hypnotics	Buspirone	1 (0.3)	Diazepam	14 (3.6)	33 (7.9)	Clomethiazole	20 (4.8)
	Flunitrazepam	5 (1.3)	Oxazepam	40 (10.4)	164 (39.4)	Melatonin	6 (1.4)
			Hydroxyzine	3 (0.8)	3 (0.7)	Midazolam	19 (4.6)
			Nitrazepam	11 (2.8)	2 (0.5)		
			Zolpidem	2 (0.5)	5 (1.2)		
			Zopiclone	34 (8.8)	113 (27.2)		
Antiepileptics	Phenobarbital	3 (0.8)	Gabapentin	1 (0.3)	8 (1.9)	Lamotrigine	4 (1.0)
	Phenytoin	4 (1.0)	Carbamazepine	23 (5.6)	6 (1.4)	Levetiracetam	17 (4.1)
			Clonazepam	2 (0.5)	1 (0.2)	Pregabalin	5 (1.2)
			Valproic acid	2 (0.5)	9 (2.2)		

Notice that patients can be prescribed several psychotropics

### Polypharmacy, PICP and PIPS

Comparison of prescribing psychotropics, including psychotropic polypharmacy, in the three nursing homes is shown in Table 2. Psychotropic polypharmacy increased significantly from 6.2% to 29.6% (p < 0.001). The use of antidepressants and anxiolytic-hypnotics increased in general, but also among patients with PIs. Table 3 shows use of combinations of psychotropics, PICP and PIPS. Prescribing of combinations of antidepressants and anxiolytic-hypnotics increased between the two years. In 2016, fourteen patients (3.4%) were prescribed combinations of two antidepressants and two anxiolytics-hypnotics. Among these, three patients were also prescribed two additional antipsychotics. Two patients were prescribed combinations of two antipsychotics and two anxiolytics-hypnotics, while one patient were prescribed a combination of two antidepressants and three anxiolytics-hypnotics. One patient was prescribed three concurrent anxiolytics-hypnotics. The increase in PICP was associated with anxiolytic-hypnotics, while a reduction in prescription of several concurrent antipsychotics or antiepileptics was observed. PIPS were reduced from 2000 to 2016, but an increase in prescription of diazepam was observed.

**Table 2:** Psychotropics prescribed to patients in 2000 and 2016 in three nursing homes with 386 and 416 patients the respective years

	2000		2016		*p-value*
	Patients n (%)	n (%)	Patients n (%)	n (%)	
All patients	386 (100.0)		416 (100.0)		
Antidepressants		97 (25.1)		170 (40.9)	< 0.001
Antipsychotics		91 (23.6)		130 (31.3)	0.015
Anxiolytics-hypnotics		102 (26.4)		268 (64.4)	< 0.001
Antiepileptics		29 (7.5)		49 (11.8)	0.042
One or more drugs	230 (59.6)		335 (80.5)		< 0.001
Antidepressants		97 (42.2)		170 (50.7)	0.045
Antipsychotics		91 (39.6)		130 (38.8)	0.856
Anxiolytics-hypnotics		102 (44.3)		268 (80.0)	< 0.001
Antiepileptics		29 (12.6)		49 (14.6)	0.494
Two or more drugs	94 (24.4)		233 (56.0)		< 0.001
Antidepressants		53 (56.4)		151 (64.8)	0.155
Antipsychotics		53 (56.4)		114 (48.9)	0.222
Anxiolytics-hypnotics		65 (69.1)		203 (87.1)	< 0.001
Antiepileptics		14 (14.9)		47 (20.2)	0.347
Three or more drugs	24 (6.2)		123 (29.6)		< 0.001
Antidepressants		17 (70.8)		97 (78.9)	0.389
Antipsychotics		17 (70.8)		72 (58.5)	0.260
Anxiolytics-hypnotics		20 (83.3)		116 (94.3)	0.062
Antiepileptics		6 (25.0)		35 (28.5)	0.730
≥ 1 interaction	63 (16.3)		146 (35.1)		< 0.001
Antidepressants		43 (68.3)		134 (91.8)	< 0.001
Antipsychotics		38 (60.3)		63 (43.2)	0.023
Anxiolytics-hypnotics		36 (57.1)		124 (84.9)	< 0.001
Antiepileptics		11 (17.5)		29 (19.9)	0.347

Chi-square test was used for comparison of proportions. Notice that patients can be prescribed several concurrent categories of psychotropics. Categories of psychotropics are associated with patients with interactions, but not necessarily involved in the interactions

**Table 3:** Prescription of combinations of psychotropic drugs, PICP and PIPS in 2000, and 2016, among patients in three nursing homes with 386 and 416 patients the respective years

	2000		2016		*p-value*
	Patients n (%)	n (%)	Patients n (%)	n (%)	
Combination of categories	80 (20.7)		210 (50.5)		< 0.001
Antidepressants + antipsychotics		26 (32.5)		62 (29.5)	0.622
Antidepressants + anxiolytics-hypnotics		32 (40.0)		127 (60.5)	0.002
Antidepressants + antiepileptics		2 (2.5)		28 (13.3)	0.005
Antipsychotics + anxiolytics-hypnotics		31 (38.8)		95 (45.2)	0.319
Antipsychotics + antiepileptics		4 (5.0)		15 (7.1)	0.605
Anxiolytics-hypnotics + antiepileptics		10 (12.5)		38 (18.1)	0.252
Potentially inappropriate combinations of psychotropic drugs (PICP)	30 (7.8)		116 (27.9)		< 0.001
Two or more antidepressants		6 (20.0)		40 (34.5)	0.153
Two or more antipsychotics		11 (37.0)		8 (6.9)	< 0.001
Two or more anxiolytics-hypnotics		7 (23.0)		86 (74.1)	< 0.001
Two or more antiepileptics*		5 (17.0)		1 (0.9)	0.001
Two or more psychotropic drugs with anticholinergic effects		3 (10.0)			
Potentially inappropriate psychotropic drugs (PIPS)	69 (17.9)		47 (11.3)		0.008
Long acting benzodiazepines					
Diazepam		14 (20.3)		33 (70.2)	
Flunitrazepam		5 (7.2)		0	
Nitrazepam		11 (15.9)		2 (4.3)	
Psychotropic drugs with anticholinergic effects					
Antidepressants					
Amitriptyline		8 (11.6)		2 (4.3)	
Clomipramine		1 (1.4)		0	
Nortriptyline		6 (8.7)		0	
Antipsychotics					
Chlorpromazine		7 (10.1)		0	
Chlorprotixene		2 (2.9)		3 (6.4)	
Levomepromazine		18 (26.1)		4 (8.5)	
Prochlorperazine		2 (2.9)		0	
Anxiolytics-hypnotics					
Hydroxyzine		3 (4.3)		3 (6.4)	

Chi-square or Fischer’s exact test was used for comparison of proportions. Notice that patients can be prescribed several concurrent categories of psychotropics. The denominator in the calculations was chosen to find the most frequent inappropriate prescribing within Combination of categories, PICP and PIPS. *Not included in definition of PICP

### PIs

The median number of PIs was 1 in both 2000 and 2016. Sixty-three patients had 94 PIs in 2000 increasing to 146 patients with 236 PIs in 2016. A range of one to four concurrent PIs in 2000 increased to one to nine in 2016. The three psychotropics most frequently prescribed to patients with PIs in 2000 were citalopram (22 patients), risperidone (16 patients) and zopiclone (13 patients). The three psychotropics most frequently prescribed to patients with PIs in 2016 were oxazepam (90 patients), mirtazapine (77 patients) and zopiclone (51 patients). Table 4 shows classification of first ranked PIs with the most frequent psychotropics involved. Mechanisms of the interactions with the associated level of documentation are described. First ranked PIs constituted 67% of all interactions in 2000 and 62.7% in 2016. First ranked PIs were increasingly pharmacodynamic with a decreased level of recommended action and documentation, but not severity. First ranked PIs involved three PIPS in 2000 and none in 2016. Antipsychotics were frequently involved in first ranked PIs in 2000 while antidepressants were frequently involved in 2016. Pharmacodynamic PIs mainly concerned risk of sedation based on theoretical documentation. Twelve PIs that should be avoided concerned risk of QT-prolongation irrespective of psychotropics involved (not only those listed in Table 4). Other PIs ranked as severe concerned risk of the serotonin syndrome. Pharmacokinetic PIs involved as expected a change in plasma levels of one or both drugs, and pharmacokinetic PIs were frequently based on study.

**Table 4:** Classification of first ranked psychotropic drug interactions, the two most frequent psychotropics involved and mechanism of interaction in 2000, and 2016, among patients in three nursing homes with 386 and 416 patients the respective years

	2000	2016		2000			2016		
	Patients n (%)	Patients n (%)	*p-value*	First (%)	Second (%)	Mechanism	First (%)	Second (%)	Mechanism
Interactions	63 (100.0)	146 (100.0)	< 0.001						
Pharmacodynamic	44 (69.8)	135 (92.5)		Citalopram (50.0)	Risperidone (22.7)	Priapism/QT-prolongation^c^	Mirtazapine (43.2)	Oxazapem (34.2)	Sedation^t^
Pharmacokinetic	19 (30.2)	11 (7.5)		Carbamazepine (42.1)	Risperidone (21.1)	Risperidone reduced^s^	Valproic acid (45.5)	Carbamazepine (36.4)	CBZ-metabolite increased^s^
Recommended action			0.006						
Avoid	7 (11.1)	5 (3.4)		Levomepromazine (71.4)	Haloperidol (57.1)	QT-prolongation^t^	Haloperidol (80.0)	Citalopram (80.0)	QT-prolongation^t^
Monitor or adjust dose	21 (33.3)	79 (54.1)		Carbamazepine (28.6)	Olanzapine (19.0)	Olanzapine reduced^s^	Mirtazapine (77.2)	Oxazepam (36.7)	Sedation^t^
Informative	35 (55.6)	62 (42.5)		Citalopram (51.4)	Risperidone (34.3)	Priapism/QT-prolongation^c^	Escitalopram (43.5)	Oxazepam (38.7)	Sedation^t^
Documentation			0.001						
Study	13 (20.7)	11 (7.5)		Carbamazepine (46.2)	Nortriptyline (23.1)	Nortriptyline reduced	Mirtazapine (54.5)	Diazepam (45.5)	CNS depression
Case	14 (22.2)	15 (10.3)		Risperidone (57.1)	Citalopram (50.0)	Priapism/QT-prolongation	Mirtazapine (40.0)	Olanzapine (40.0)	Sedation
Theoretical	36 (57.1)	120 (82.2)		Citalopram (38.9)	Levomepromazine (19.4)	QT-prolongation	Mirtazapine (44.2)	Oxazepam (40.0)	Sedation
Severity			0.222						
Severe	35 (55.5)	64 (43.8)		Citalopram (42.9)	Risperidone (40.0)	Priapism/QT-prolongation^c^	Mirtazapine (43.8)	Escitalopram (37.5)	Serotonine syndrome^t^
Moderate	27 (42.9)	81 (55.5)		Citalopram (25.9)	Zopiclone (22.2)	Sedation^t^	Oxazepam (60.5)	Mirtazapine (45.7)	Sedation^t^
Mild	1 (1.6)	1 (0.7)		Zopiclone (100.0)	Carbamazepine (100.0)	Sedation^t^	Zopiclone (100.0)	Carbamazepine (100.0)	Sedation^t^

Chi-square or Fischer’s exact test was used for comparison of proportions. c; Case, s; Study, t; Theoretical. Levomepromazine and nortiptyline are PIPS; potentially inappropriate psychotropic substances. Combination of levomepromazine and haloperdiol, and combination of mirtazapine and escitalopram, are both PICP. Notice that patients can have several concurrent psychotropic drug interactions. CBZ; carbamazepine

### Outcomes and predictors

The variance inflation factors were < 2.0 and tolerance values were > 0.5 for all predictors in multicollinearity analysis in both 2000 and 2016. Predictors for having PIs from the univariate logistic regression analysis with p < 0.25 that could be included in the multivariable logistic regression analysis was number of psychotropics and prescription of antidepressants, antipsychotics (only in 2016), and anxiolytics-hypnotics. In multivariable analysis only number of psychotropics was predictive of having PIs in 2000. In 2016, number of psychotropics and prescription of antidepressants were predictive of having PIs (Table 5).

**Table 5:** Univariate and multivariate logistic regression analyses of predictive variables for having psychotropic interactions in 2000, and 2016, among patients in three nursing homes with 386 and 416 patients the respective years

Predictive variables	2000				2016			
	n = 386				n = 416			
	Unadjusted model		Adjusted model		Unadjusted model		Adjusted model	
	OR (95% CI)	*p-value*	OR (95% CI)	*p-value*	OR (95% CI)	*p-value*	OR (95% CI)	*p-value*
Gender	1.60 (0.59–4.31)	0.358			1.26 (0.72–2.18)	0.425		
Age	0.99 (0.94–1.05)	0.741			0.99 (0.96–1.02)	0.457		
Number of psychotropic drugs	4.28 (1.25–14.64)	0.021	6.06 (1.61–22.81)	0.008	2.95 (1.97–4.42)	< 0.001	2.58 (1.43–4.63)	0.002
Prescribed antidepressants	4.52 (1.80–11.34)	0.001	3.18 (1.12–9.04)	0.030	45.98 (20.80–101.67)	< 0.001	29.00 (12.23–68.77)	< 0.001
Prescribed antipsychotics	1.62 (0.68–3.86)	0.274			0.54 (0.31–0.92)	0.023	0.61 (0.24–1.51)	0.282
Prescribed anxiolytics-hypnotics	0.09 (0.02–0.42)	0.002	0.07 (0.02–0.35)	0.001	0.57 (0.24–1.35)	0.200	0.51 (0.15–1.78)	0.293
Prescribed antiepileptics	1.97 (0.51–7.67)	0.326			0.95 (0.49–1.84)	0.879		

*OR* Odds ratio, *CI* Confidence interval

## Discussion

The present study found a significant change in inappropriate prescribing of psychotropics in three Norwegian nursing homes audited 16 years apart. Using prescribing quality indicators we observed that antidepressants and anxiolytics-hypnotics were more frequently inappropriately prescribed in the present decade. In contrast, antipsychotics and antiepileptics were less frequently inappropriately prescribed compared to the previous decade. Furthermore, the prescribing quality indicators also showed that inappropriate prescribing of psychotropics in 2016 was associated with increased polypharmacy and PIs, mainly pharmacodynamic, with a low level of documentation. In the following, the results are discussed with relevance for medical informatics.

The increase in PIs was associated with prescribing of antidepressants. The proportion of patients with PIs in 2016 was almost doubled compared to 2000, although contraindicated PIs were reduced. Among first ranked PIs, an increased proportion was pharmacodynamic with mechanisms mainly based on theoretical documentation. Only nine of all 179 (5 %) pharmacodynamic first ranked PIs in the present results were based on studies. High quality documentation to support the existence of many drug interactions is still lacking [20, 21]. Low quality documentation could result in failure to recognise drug interactions (sensitivity) or risk of misclassification (specificity). Poor inter-rater agreement was observed in a small study with two groups of eight general practitioners who reviewed 100 randomly selected drug interactions according to available documentation and their clinical experience. In 97 out of 100 interactions there was at least some disagreement on whether the interaction was clinically significant [22].

The most serious PIs concerned QT-interval prolongation and the serotonin syndrome. However, both conditions are associated with a low level of documentation and controversy. QT-interval prolongation is regarded as a potential serious effect of psychotropics with risk of sudden cardiac death [23]. However, drugs that prolong the QT-interval range from having potent torsadogenic activity to no proarrhythmic action and even antiarrhythmic effects [24]. Two recent studies found no correlation between the number of QT-prolonging drugs in a patient and degree of corrected QT (QTc) prolongation [25, 26]. Several authors state that QT-prolongation is a bad surrogate for serious ventricular arrhythmias [24, 27, 28]. The serotonin syndrome is a potentially fatal and largely avoidable adverse drug reaction caused by serotonergic drugs [29]. Associations and cautions concerning the rare but serious serotonin syndrome have proliferated in the medical literature [29]. Clinicians prescribing serotonin reuptake inhibitors (SSRIs) can expect to be warned about 1000 interaction drugs where the majority have little or no evidence of an interaction.

Risk of sedation by combining two or more psychotropics was the most frequent mechanism among all the first ranked PIs, but also among all the other PIs in the material irrespective of year. A study among 455 residents in a long-term care setting found that the alerts most often triggered by prescribing were risk of sedation [30]. However, drug interaction alerts are frequently overridden. A hospital study found that 93% of alerts from an electronic medical record and clinical decision system were overridden with no difference between experienced or unexperienced physicians [31]. The most common overall reason for overriding alerts was, according to another study, the patient had previously tolerated the medication [32]. A potential relevant observation in the present results is that there were no stratification to age, gender or dose when examining the pharmacodynamic PIs [19].

Pharmacokinetic PIs in drug interaction databases are frequently supported by studies. In the present results, 15 of the 30 pharmacokinetic PIs (50.0%) were based on studies. Studies and case reports provide description of clinical and laboratory monitoring and management options with dose adjustments that can be included in prescribing tools. It is plausible the reduction in pharmacokinetic PIs from 2000 to 2016 could reflect more awareness among prescribers due to improved quality of the documentation. To support this, we observed a reduction in prescription of drugs with risk of pharmacokinetic interactions like carbamazepine, paroxetine, phenobarbital and phenytoin between 2000 and 2016.

The general increase in use of psychotropics from 2000 to 2016 mainly concerned anxiolytics-hypnotics and antidepressants with a substantial increase in psychotropic polypharmacy and PICP. Psychotropic polypharmacy was found in 14.5% of residents on a regular basis in recent study of 881 patients from 30 Norwegian nursing homes. In that study, a higher risk was found in female residents, residents residing in long-term wards, and residents with the best performance in activities of daily living [15]. In the present study, a high proportion of polypharmacy (30%) may in part be explained by including drugs on demand. Yet, the reduction in PIPS we observed from 2000 to 2016 is not necessarily explained by improved medical informatics and increased awareness. Several PIPS are no longer licensed in Norway and have been replaced by new psychotropics. Prescribing in 2016 of diazepam and clomethiazole to nearly 8 and 5% of the patients respectively, was surprising since these drugs are not recommended in this population [15]. However, nearly 50% of diazepam was prescribed as needed for epileptic seizures, and the other 50% were equally divided between regularly prescribed and as needed. The other prescriptions was PIPS with anticholinergic effects have been associated with risk of cognitive decline. However, a single blind randomized trial in Norway found no improvements in cognitive function eight weeks after reducing anticholinergic drugs in patients at nursing homes [33]. Furthermore, it has been argued that patients with cognitive decline could have symptoms that result in anticholinergic prescriptions [34].

Increase in PICP in 2016 was related to combinations of zopiclone and oxazepam, both frequently prescribed drugs. Anxiolytics and hypnotics used routinely or as needed can lead to polypharmacy, potentially harmful drug interactions, and total drug doses exceeding the maximum recommended [18]. Less rational combinations like two antidepressants and two anxiolytics-hypnotics were also observed. Increased use of antidepressants and anxiolytics-hypnotics has been reported in several recent studies in Norwegian nursing homes [35–37]. A recent study found a significant decrease in prescribing of antipsychotic drugs to patients with mild dementia between 2004 and 2014 with no change in prescription of other psychotropics [38]. In the present results the proportion of patients with antipsychotics did not increase significantly, and was reduced among patients with PIs and in particular among patients with PICP from 2000 and 2016. A possible explanation is that reduced prescribing of antipsychotic drugs for behavioural symptoms is compensated by increased prescription of other categories of psychotropics [35, 39]. Increased risk of cerebrovascular adverse effects and mortality in elderly patients has been associated with conventional and atypical antipsychotics in several studies [40, 41]. U.S Food and Drug Administration warnings of increased risk of death associated with atypical (in 2005) and conventional (in 2009) antipsychotics in patients with dementia gained attention in Norwegian drug information sources. Stabilization or a reduction in prescribing of antipsychotics in nursing homes suggests that medical informatics could have some success in increasing awareness for this category of psychotropics in nursing homes. However, antipsychotic drug use was not associated with long-term mortality risk among 1163 patients over a 75-month follow-up period in Norwegian nursing home patients [42].

The importance of antidepressants and anxiolytics-hypnotics for inappropriate prescribing in the present decade could be related to several factors. The use of these psychotropics for behavioural symptoms to compensate for reduced prescribing of antipsychotics has been mentioned above. Changes in organisation, staff expectations, drug safety perception, drug licence policy, and demographic and clinical characteristics of patients are among other possible factors. However, from a medical informatics perspective prescribing to nursing home patients should focus on drug safety irrespective of patient factors. In our opinion, the more ill patients should at least avoid inappropriate drugs and drug combinations. Notably, prescribing quality indicators of relevance in 2000 could be redundant in 2016 due to a change in clinical practice and available drugs on the market. Furthermore, low specificity (e.g. it is rational to use of diazepam in epileptic seizures) or a low level of documentation (e.g. pharmacodynamic PIs) could contribute to ignorance of alerts.

### Strength and Limitations

Classification of PIs in both 2000 and 2016 was performed using a single subscription database updated in 2017. However, this database scores statistically high with regard to ownership, classification of interactions, primary information sources and staff qualification in comparison with open access drug interaction databases [14]. Prescribing indicators like PICP and PIPS have been used in several research studies [12, 43]. The nursing homes included in the present study had access to modern prescribing tools. From 2007/2008, two of the nursing homes had access to pilot a computerized decision support system called the Geriatric Basis Dataset (GBD), which included the interaction tool in the Norwegian Pharmaceutical Product Compendium, while the third nursing home gained access to pilot the system in 2012. Furthermore, GBD include The Norwegian General Practice (NORGEP) criteria for prescribing to elderly > 70 years [43].

Limitations include the theoretical approach of this small study, lack of clinical information, lack of information about other drugs, and information concerning organisation and staff within the institutions. The respective patient populations in 2000 and 2016 could be quite different since the nursing homes had more specialized units in 2016. Increased comorbidity, dementia and polypharmacy among the patients could warrant more or different psychotropics. Only one drug interaction database was used for classification of clinically relevant PIs while it is well known that drug interaction databases lacks consistency [20, 21]. Furthermore, the most important PI in each patient (about 63-67% of all PIs) was used to describe qualities of psychotropic interactions, while use of other databases could provide different ranking. Only potential PIs are described, and all prescriptions (regular and on demand) were included which may increase the risk of overestimating daily use of psychotropics. Anxiolytics and hypnotics as a single category could also overestimate PICP.

## Conclusions

In this small Norwegian study, we observed an increasing importance of antidepressants and anxiolytics-hypnotics for inappropriate prescribing of psychotropics in nursing homes in 2016 compared to 2000. Prescribing quality indicators found that the inappropriateness of psychotropics in 2016 were associated with mainly pharmacodynamic interactions, lack of good documentation, and controversial assertions. The relevance of these qualities for the persistent problem of inappropriate prescribing of psychotropics in nursing homes, and if this reduces the impact of decision support systems, is of interest to study. Furthermore, our identification of the main drug categories for inappropriate prescribing in nursing homes in this decade could be used for planning specific medical informatics-based approaches in these institutions. A suggestion would be to address use of antidepressants and anxiolytic-hypnotics in Norwegian nursing homes through an academic detailing program. Furthermore, we suggest a revision of prescribing quality indicators in cooperation with the prescribers themselves.

## Abbreviations

ATC: Anatomical Therapeutic Chemical classification system
GBD: Geriatric Basis Dataset
NORGEP: The Norwegian General Practice criteria for prescribing to elderly > 70 years
PICP: Potentially inappropriate combinations of psychotropics
PIPS: Potentially inappropriate psychotropic substances
PIs: Psychotropic interactions
SSRIs: Selective serotonin reuptake inhibitors
